# Colorectal Cancer Brain Metastasis With Concomitant KRAS and BRAF Mutations: A Case Report

**DOI:** 10.7759/cureus.68975

**Published:** 2024-09-09

**Authors:** Alexandra Vesa, Octavian Maghiar, Ottó Molnar, Ovidiu Pop, Adrian Maghiar

**Affiliations:** 1 Morphological Sciences, University of Oradea, Faculty of Medicine and Pharmacy, Oradea, ROU; 2 Surgical Sciences, University of Oradea, Faculty of Medicine and Pharmacy, Oradea, ROU; 3 Doctoral Studies (Biomedical Science), University of Oradea, Faculty of Medicine and Pharmacy, Oradea, ROU; 4 Surgery Sciences, University of Oradea, Faculty of Medicine and Pharmacy, Oradea, ROU

**Keywords:** braf mutation, brain metastasis, colorectal cancer, concomitant mutations, kras

## Abstract

Colorectal cancer (CRC) brain metastasis (BM) is a rare but aggressive manifestation of the disease, with poor prognosis and limited treatment options. Although brain metastases are more commonly associated with primary tumors located in the lung, skin, and breast, their occurrence in colorectal cancer is uncommon. Genetic mutations are highly important in tumor progression, and mutations in KRAS and BRAF genes are key drivers in colorectal cancer. However, the concurrent presence of both mutations is exceedingly rare. This case report presents a unique instance of colorectal cancer brain metastasis harboring both KRAS and BRAF mutations, highlighting its clinical significance and therapeutic challenges.

We present the case of a 62-year-old male patient diagnosed with brain metastasis (in the cerebellum and right parietal lobe) who presented to the hospital with neurological symptoms. He underwent a CT imaging investigation that revealed multiple tumors. Subsequent biopsies confirmed the diagnosis of brain metastasis, with histological characteristics consistent with colonic adenocarcinoma. Tests also revealed aberrant expression of both KRAS and BRAF mutations.

This case highlights the importance of considering brain metastases in colorectal cancer patients due to their detrimental effects on prognosis and survival rates. Additionally, the simultaneous presence of BRAF and KRAS mutations, in this case, adds an extra layer of complexity and severity.

## Introduction

Colorectal cancer (CRC) incidence has been progressively increasing worldwide, especially in developing nations adopting a "Western" lifestyle [1]. CRC is a significant global health concern, ranking as the third most commonly diagnosed cancer in both men and women worldwide, with a total of 1,931,590 new cases reported in 2020, representing approximately one in ten cancer cases and deaths [2,3]. While it is highly treatable and preventable when detected early, it becomes more challenging to manage when left undiagnosed or untreated.

Brain metastases (BM) from colorectal cancer are relatively uncommon but progress quickly, leading to a poor prognosis and reduced quality of life. These metastases typically arise from primary tumors in the lung, skin, breast, or genital area, making brain involvement especially unusual [4-7].

The development of colorectal cancer occurs in well-defined stages linked to specific genetic and epigenetic changes in various oncogenes and tumor suppressor genes. The KRAS gene, present in about 50-60% of colorectal tumors, typically carries a missense mutation. The BRAF gene is found in 5-10% of tumors. While previously believed to be mutually exclusive, several cases of concomitant KRAS and BRAF mutations in colorectal cancer have been reported, occurring in less than 0.001% of cases, demonstrating that the presence of multiple variations in the BRAF and RAS pathways can occur, albeit rarely [8-10]. Furthermore, the presence of both KRAS and BRAF mutations is associated with more aggressive disease [11].

## Case presentation

A 61-year-old male patient presented to the emergency department with diffuse abdominal pain and constipation. A colonoscopy revealed a stenotic sigmoid tumor, resulting in sigmoidectomy surgery with a colostomy. Microscopic examination confirmed a moderately differentiated intestinal adenocarcinoma (pT3N2b) with peritumoral lymph node involvement. Blood tests indicated mild iron-deficiency anemia and elevated carcinoembryonic antigen (CEA) levels. Imaging showed no metastases at this point. The patient underwent oncological treatment, including eight cycles of FOLFOX, with good tolerance and no significant side effects. Five months post-surgery, multiple polyps were discovered in the ascending colon during a control colonoscopy, diagnosed as tubulo-villous adenoma with high-grade dysplasia.

**Figure 1:**
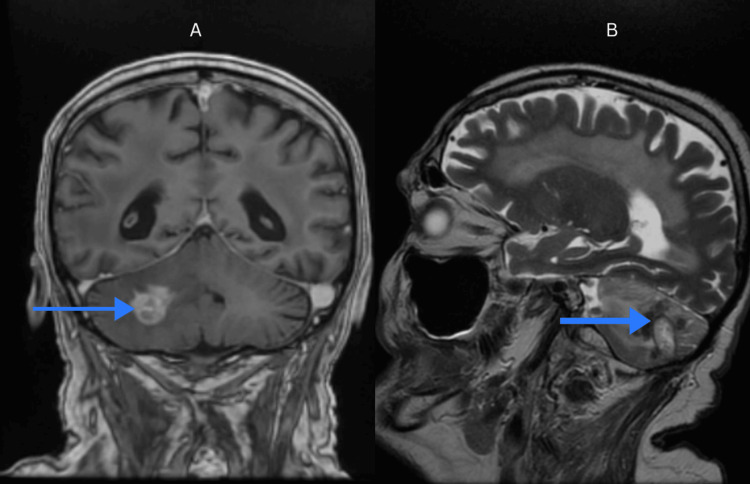
MRI sagittal scan. MRI brain demonstrating at the level of the right cerebellar hemisphere, a native hypodense area (blue arrow), with a heterogeneous appearance, with some hyperdense images and intracranial aerocele, with a compressive effect on V4.

**Figure 2:**
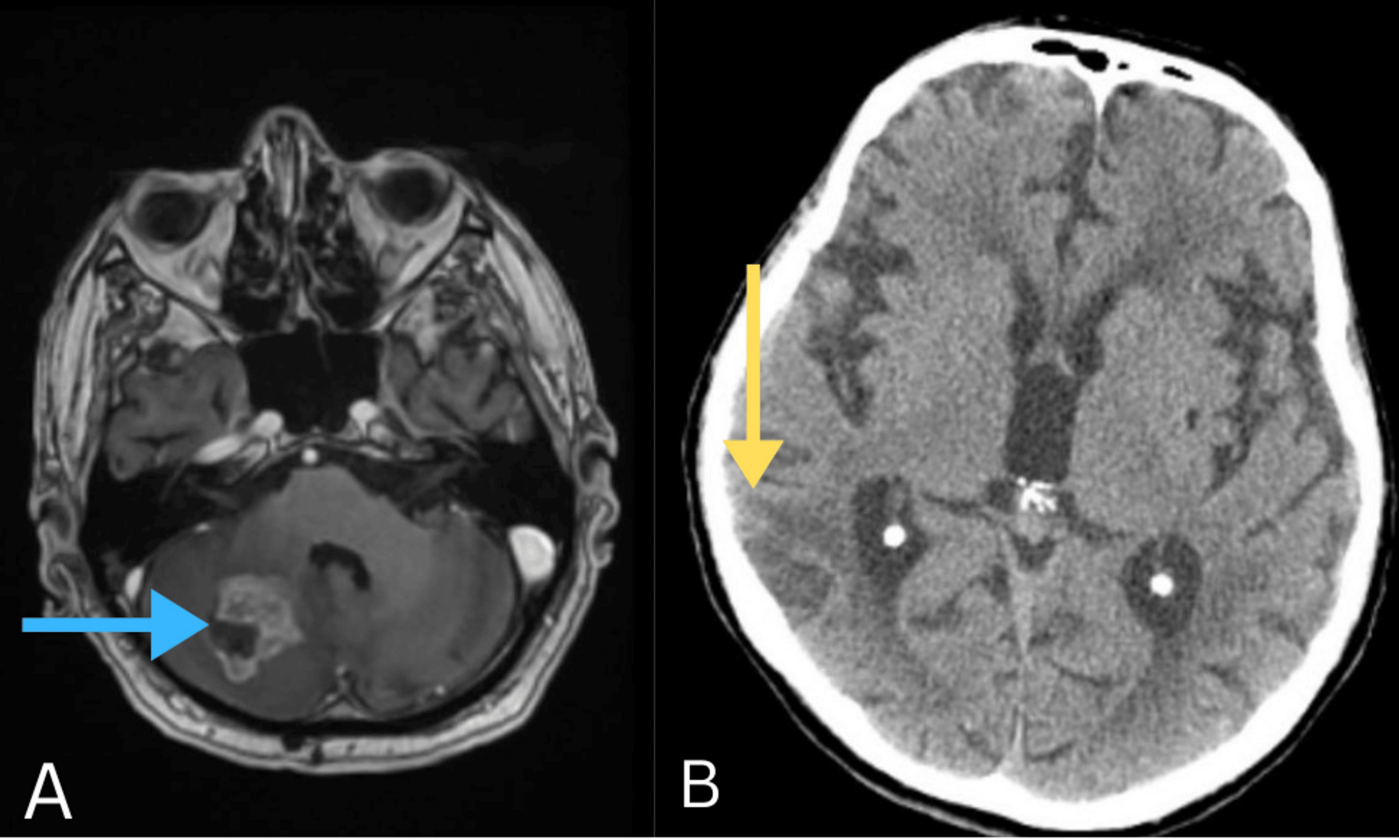
MRI an CT scan. (A) MRI axial 3DT1: the presence of brain metastasis in the right cerebellar hemisphere (blue arrow); (B) CT axial: at the level of the right parietal lobe, a dense heterogeneous mass with a perilesional mass effect and peripheral edema in the vicinity (yellow arrow).

Fifteen months after surgery, the patient presented to the emergency department with neurological symptoms, including headache, dizziness, confusion, diplopia, balance issues, and walking difficulties. A complete blood count showed leukocytosis with neutrophilia and moderate anemia. CEA was also increased with a value of 17 ng/mL. Further radiological examinations revealed a right cerebellar tumor of 3.3/1.6 cm plus a right parietal formation of 4.3/2.6 cm (shown in Figures 1-2).

**Figure 3:**
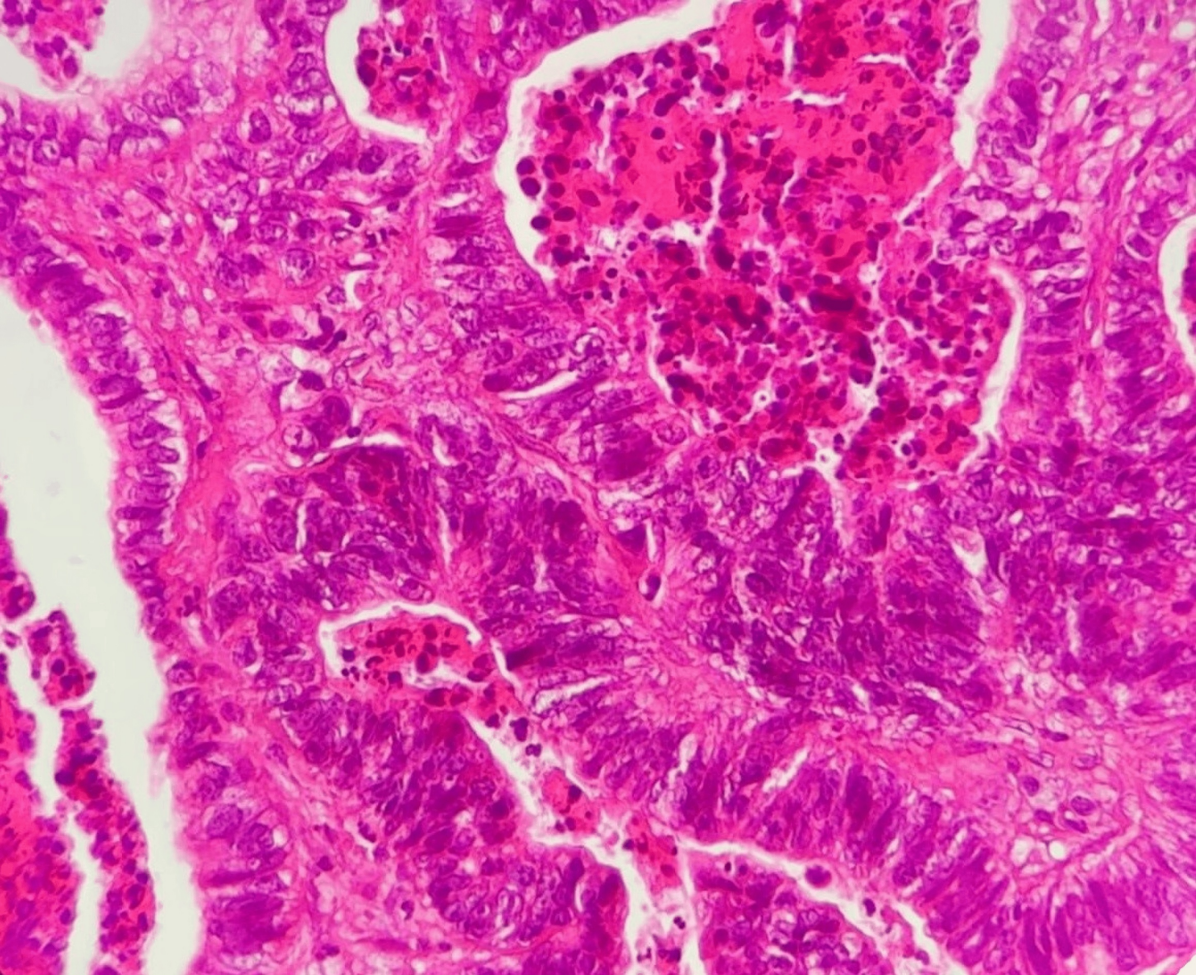
HE ×40. Light microscopic picture of metastatic colorectal adenocarcinoma-cerebellar. Tall malignant columnar cells lining large irregular glands with cellular and nuclear atypia; dirty necrosis present in the lumen of the glands.

**Figure 4:**
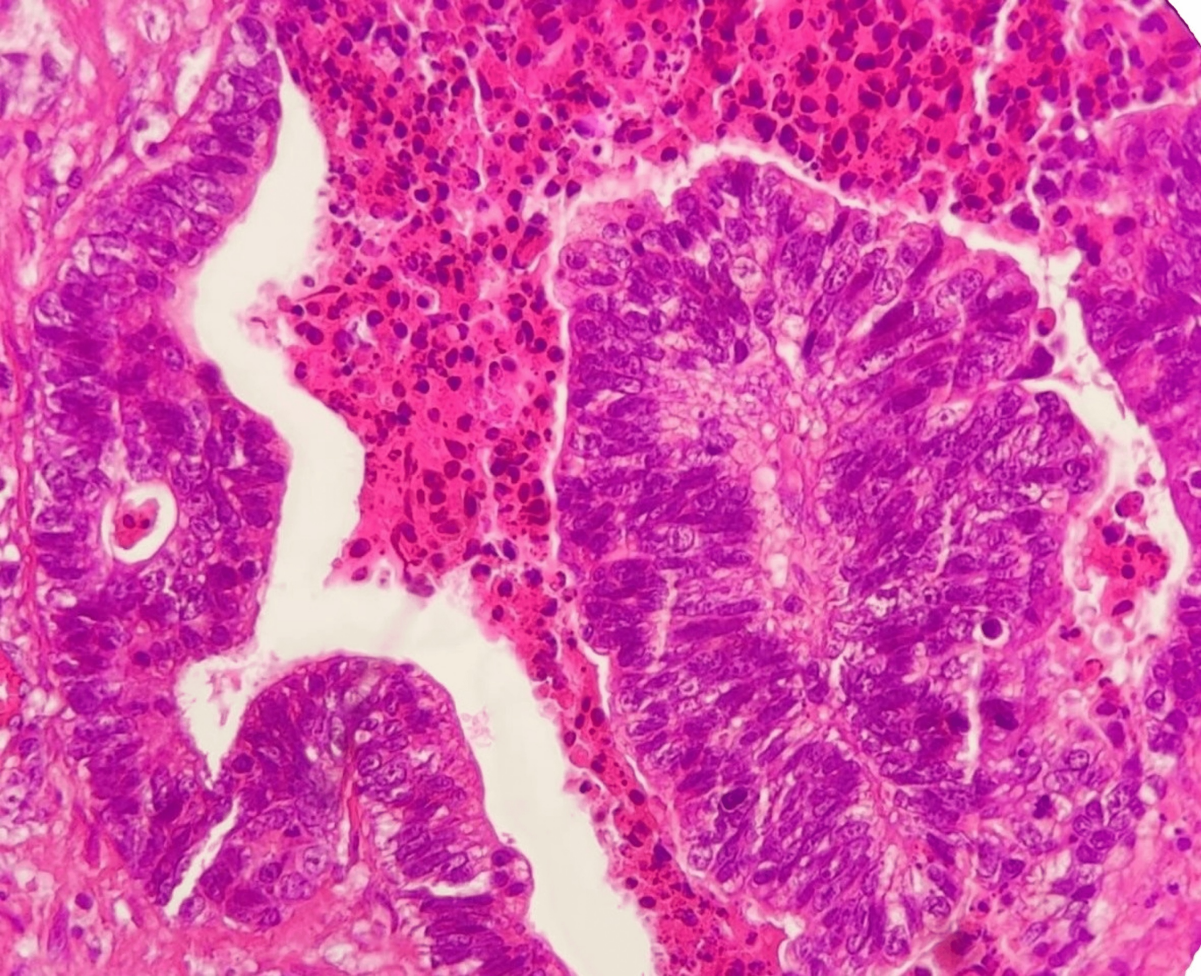
HE ×40. Light microscopic picture of metastatic colorectal adenocarcinoma-cerebellar.

The biopsy reveals the presence of a cellular proliferation infiltrating the brain tissue composed of glands of different shapes and sizes, covered by tall atypical cells arranged in several rows, with moderate to marked nuclear pleomorphism, hyperchromatic nuclei, and atypical mitoses present. Dirty necrosis is observed in the glandular lumen. These aspects correlate with the diagnosis of intestinal adenocarcinoma metastasis of colonic origin (shown in Figures 3-4).

Genetic analysis shows both KRAS (KRAS: c.34G>A (p.(Gly12Ser))) and BRAF mutant (c.1799T>A (p.(Val600Glu))). Moreover, the immunohistochemical study of the tumor sample showed intact nuclear expression of MLH-1, MSH-2, and MSH-6, consistent with microsatellite stability. The patient initially underwent one session of radiotherapy; however, due to a rapid decline in their clinical condition and a performance status of 4 (according to the ECOG scale), the decision was made to discontinue further radiation therapy. Given the patient's poor functional status and limited capacity to tolerate aggressive treatments, the multidisciplinary team concluded that the risks of continuing radiotherapy or initiating chemotherapy outweighed the potential benefits. As a result, we shifted the focus to supportive care, prioritizing the patient’s comfort and quality of life. This supportive care included pain management, symptom control, nutritional support, and psychological counseling in alignment with palliative care principles. The decision to avoid chemotherapy or targeted agents such as bevacizumab was based on the patient's frailty, advanced disease state, and poor performance status, which precluded further active oncologic treatments.

**Figure 5:**
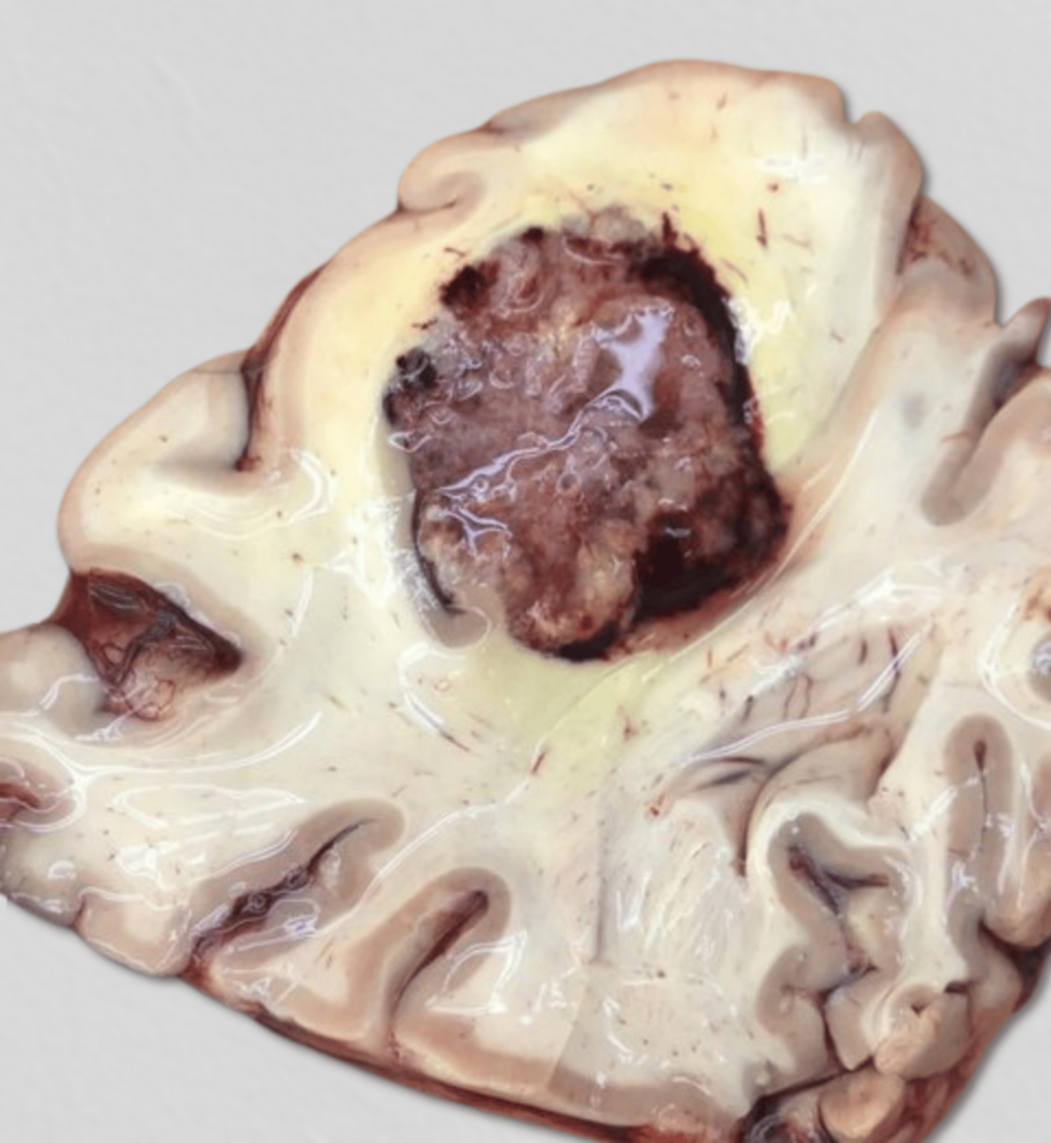
Gross appearance of the right parietal lobe mass.

**Figure 6:**
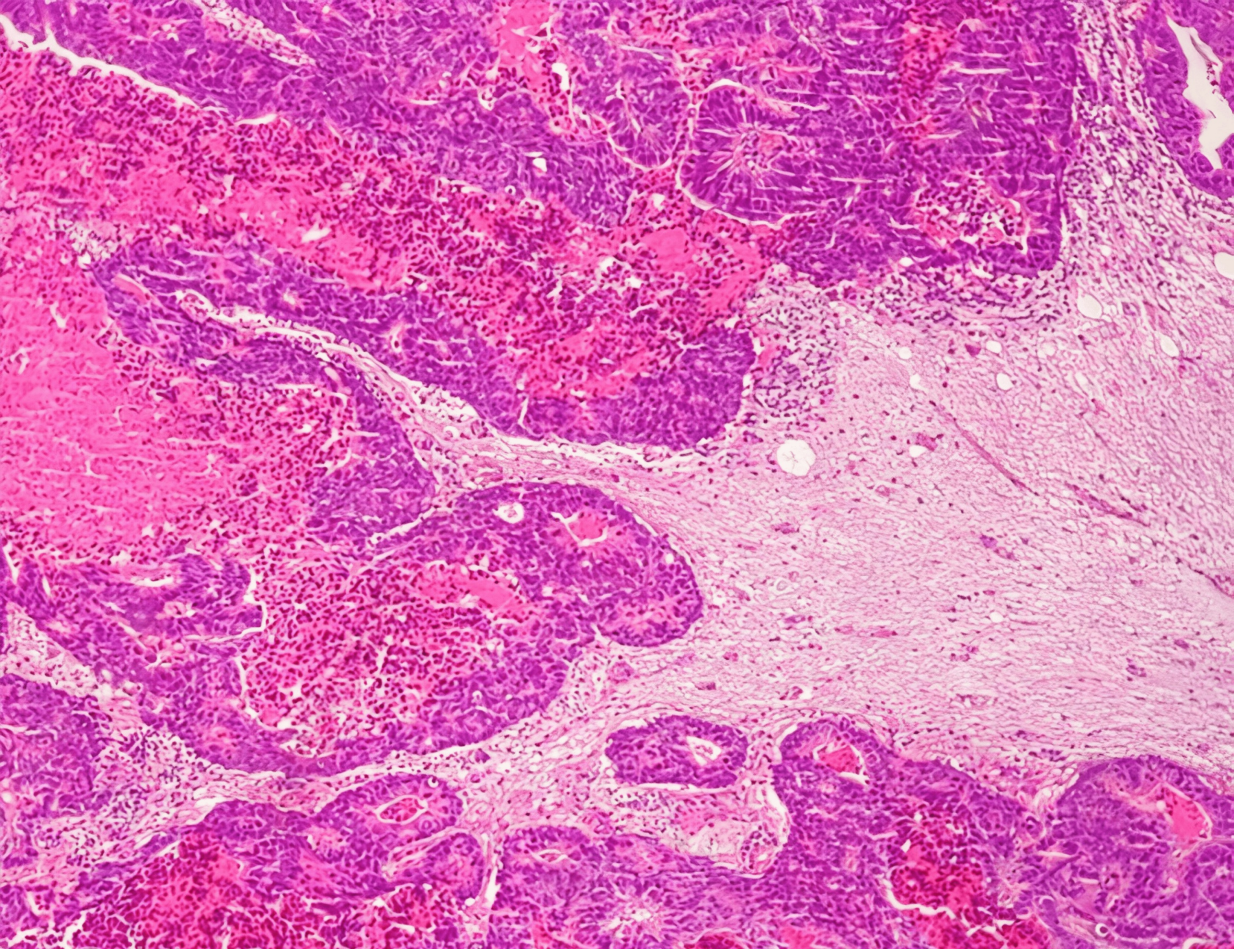
HE ×4. Light microscopic image of metastatic colorectal adenocarcinoma – right parietal lobe.

Despite treatment, the patient's condition deteriorated, and he passed away two months after the brain metastasis diagnosis, with no evidence of liver or lung metastasis at that time. Following the patient's death, a post-mortem examination was conducted, during which detailed images were obtained to further analyze the metastatic spread (Figures 5-6).

## Discussion

Brain metastasis from colorectal cancer represents a rare but clinically significant phenomenon that poses unique challenges in both diagnosis and management. Colorectal cancer itself is a prevalent malignancy, primarily affecting the colon and rectum, but its metastatic spread to the brain is relatively uncommon.

There is no doubt that the liver and the lung are the most typical metastatic sites for CRC patients [11]. The reported occurrences of brain metastases range from 0.1% to 11.5%, with an average incidence of 2.1% [6]. In a study performed by Yang et al. from a total of 170,793 cases diagnosed with CRC during 2010-2013, only 401 patients were identified with brain metastasis (0.23%) [12,13]. More often, brain metastasis develops from tumors with a primary origin located at the lung, breast, skin, and genital level. The data from the literature show several cases describing cerebral metastasis of colorectal cancer, but so far, we have not found any cases regarding BM from CRC with both mutations present. We recently performed a retrospective observational study that included 118 patients diagnosed with colorectal cancer, and the incidence of brain metastasis was 1.69% (n = 2); both cases had concomitant KRAS and BRAF mutations present. The median age at the diagnosis of brain metastasis in patients diagnosed with CRC has been reported to range from 56 to 73 years. Comparable to the data offered by the literature, our patient was 62 years old at the time the brain metastasis was diagnosed.

The pattern of metastatic spread seems to be affected by the presence of a KRAS mutation [14]. After a diagnosis of metastatic illness, the RAS mutation is an independent predictor of metastasis to the lung, bone, and brain and is associated with a noticeably greater cumulative incidence of these secondary tumors. In two studies, it was established that RAS mutations are substantially related to BM [15]. In many clinical trials, the BRAF mutation has been discovered to represent a poor prognostic factor, plus it was also suggested that it decreases the incidence of liver-limited metastasis. BRAF mutant CRC more frequently exhibits unfavorable histological characteristics such as lymphatic invasion, a high tumor budding rate, perineural invasion, and increased lymph node metastases (our patient had lymph node metastasis in all 11 regional lymph nodes identified) [16].

Additionally, it is well known that the coexistence of RAS/BRAF mutations is linked to a worse clinical outcome, a more advanced tumor stage, and distant or loco-regional metastases in patients with CRC [10]. In the case presented by us, the patient is diagnosed from the beginning with an advanced-stage disease (IIIC), has a short disease-free period (15 months), and the newly appearing metastases are in an unusual place for colorectal cancer (brain).

Patients with BM have the lowest median survival rates among those with metastatic colorectal cancer (0.4-7.4 months versus 21-30 months) and even lower compared to brain metastasis from other sites like lung, breast, or skin tumors [17-19]. In our case, the patient died two months after the diagnosis of brain metastasis. The surprising particularity of the case comes from the fact that there is a simultaneous KRAS and BRAF mutation, which, according to data from the literature, is a very rare finding. Evaluation of the co-occurrence of these mutations should be taken into account in addition to single gene changes in MAPK signaling [20]. The simultaneous existence of the two mutations gives the patient a much harsher and worse prognosis compared to patients with KRAS and BRAF wild-type, and we wanted to highlight the importance of considering brain metastases in colorectal cancer patients and the potential impact of rare mutations on disease progression and prognosis.

## Conclusions

In conclusion, this case report highlights a rare and aggressive presentation of colorectal cancer with brain metastasis harboring concomitant KRAS and BRAF mutations. This unique genetic profile poses significant challenges for treatment, as it is associated with a poorer prognosis and limited therapeutic options. The case emphasizes the need for further research into targeted treatment approaches for patients with such rare genetic mutations, particularly in metastatic settings. A deeper understanding of these mutations and the development of novel therapies could lead to improved outcomes for this subset of patients, who currently face limited treatment avenues and a rapidly progressing disease course.
